# The Effect of Different Head Movement Paradigms on Vestibulo-Ocular Reflex Gain and Saccadic Eye Responses in the Suppression Head Impulse Test in Healthy Adult Volunteers

**DOI:** 10.3389/fneur.2021.729081

**Published:** 2021-09-22

**Authors:** Dmitrii Starkov, Bernd Vermorken, T. S. Van Dooren, Lisa Van Stiphout, Miranda Janssen, Maksim Pleshkov, Nils Guinand, Angelica Pérez Fornos, Vincent Van Rompaey, Herman Kingma, Raymond Van de Berg

**Affiliations:** ^1^Division of Balance Disorders, Department of Otorhinolaryngology and Head and Neck Surgery, Maastricht University Medical Center, Maastricht, Netherlands; ^2^Faculty of Physics, Tomsk State Research University, Tomsk, Russia; ^3^Department of Methodology and Statistics, Care and Public Health Research Institute (CAPHRI), Maastricht University, Maastricht, Netherlands; ^4^Service of Otorhinolaryngology Head and Neck Surgery, Department of Clinical Neurosciences, Geneva University Hospitals, Geneva, Switzerland; ^5^Faculty of Medicine and Health Sciences, University of Antwerp, Antwerp, Belgium; ^6^Department of Otorhinolaryngology and Head and Neck Surgery, Antwerp University Hospital, Edegem, Belgium

**Keywords:** vestibular ocular reflex, video head impulse test (vHIT), suppression head impulse paradigm, active head impulse, passive head impulse, inward head impulse, outward head impulse

## Abstract

**Objective:** This study aimed to identify differences in vestibulo-ocular reflex gain (VOR gain) and saccadic response in the suppression head impulse paradigm (SHIMP) between predictable and less predictable head movements, in a group of healthy subjects. It was hypothesized that higher prediction could lead to a lower VOR gain, a shorter saccadic latency, and higher grouping of saccades.

**Methods:** Sixty-two healthy subjects were tested using the video head impulse test and SHIMPs in four conditions: active and passive head movements for both inward and outward directions. VOR gain, latency of the first saccade, and the level of saccade grouping (PR-score) were compared among conditions. Inward and active head movements were considered to be more predictable than outward and passive head movements.

**Results:** After validation, results of 57 tested subjects were analyzed. Mean VOR gain was significantly lower for inward passive compared with outward passive head impulses (*p* < 0.001), and it was higher for active compared with passive head impulses (both inward and outward) (*p* ≤ 0.024). Mean latency of the first saccade was significantly shorter for inward active compared with inward passive (*p* ≤ 0.001) and for inward passive compared with outward passive head impulses (*p* = 0.012). Mean PR-score was only significantly higher in active outward than in active inward head impulses (*p* = 0.004).

**Conclusion:** For SHIMP, a higher predictability in head movements lowered gain only in passive impulses and shortened latencies of compensatory saccades overall. For active impulses, gain calculation was affected by short-latency compensatory saccades, hindering reliable comparison with gains of passive impulses. Predictability did not substantially influence grouping of compensatory saccades.

## Introduction

The peripheral vestibular system is the part of the inner ear, and it includes three semicircular canals and two otolith organs. The semicircular canals detect 3D head rotations and induce eye movements in the opposite direction of head rotation. This is the angular vestibulo-ocular reflex (VOR) (1). The VOR enables gaze stabilization during head movements. In case of reduced or absent function of the semicircular canals, the VOR is affected, which might cause oscillopsia, a symptom of blurred vision during head movements (2).

The function of all three semicircular canals can be assessed in the high-frequency domain using the video head impulse test (vHIT) (3). During this test, while the subject is fixating a visual target, an examiner rotates the subject's head with a brisk, small amplitude and high angular velocity. Such a head turn delivered by the examiner is called a “head impulse.” The head and eye movements are then recorded simultaneously by a device, which is either a pair of goggles mounted with a high-speed infrared camera or only a remote camera fixated in front of the subject. In the first case, a camera mounted on a goggle frame detects eye movements, while gyroscopes positioned on the same frame record head movements. In the second case, both eye and head movements are derived from images recorded by the camera (4). The ratio of eye to head angular velocity, called gain, is used as a parameter to assess the VOR. In healthy subjects, this ratio is close to 1.

Two different testing paradigms exist in vHIT: the head impulse paradigm (HIMP) and the suppression HIMP (SHIMP). They differ with respect to the target of fixation. In HIMP, the target is fixated with respect to the earth at a distance of 1.5–2 m from the test subject (3). In SHIMP, the target is projected on a wall in front of the subject, and it moves synchronously with the head of the test subject (5).

In HIMP, healthy subjects are able to keep their gaze on the target during the head impulse due to the VOR. Patients with an impaired VOR are not able to keep their eyes on the target and have to produce fast eye movements to reposition the eyes on the target: the catch-up saccades. In contrast to HIMP, healthy subjects in SHIMP have to produce saccades since the VOR drives the eyes in the opposite direction of the head impulse, while the target is moving synchronously with the head. Patients with an absent VOR do not have to produce saccades during SHIMP, since their eyes are already moving along with the head during the head impulse, keeping them on the target. Both paradigms are able to indicate loss of semicircular canal function (3, 5), although SHIMP might better indicate residual vestibular function (6) and might more reliably facilitate gain calculation in patients with severe loss of vestibular function. Since in SHIMP physiological saccadic eye movements appear in healthy subjects (5), SHIMP is the paradigm of choice in investigating saccadic eye responses during head impulses in healthy subjects.

The head impulse itself can be performed in different ways, which might have an effect on the predictability of the vHIT. These head impulse variations mainly include differences in direction (inward vs. outward direction) and type of movements, which are either delivered by the examiner (passive) or produced by test subjects themselves (active). Since subjects know the direction of inward head movements, this makes inward impulses more predictable than outward impulses (7). Since subjects know both timing and direction of active head impulses, this makes active impulses more predictable than passive head impulses (8).

Although the consequences of different head impulses for outcome measures in vHIT have not been well established, recent studies have reported effects of predictability on gain values and saccadic eye responses. Conflicting evidence exists about the effect of predictability on VOR gain. VOR gain was found to be decreased in inward, more predictable head movements in HIMP. This could involve impulses to both directions, or only to the contralesional side in patients with unilateral vestibulopathy (7, 9, 10). However, another study did not find any difference between passive inward and outward head impulses in healthy subjects (11).

Regarding active, more predictable head movements, VOR gain increased in patients with unilateral vestibulopathy (8, 12) but remained unchanged in healthy subjects (12), as compared with passive head movements. Saccade latency was shorter in more predictable passive inward rather than in passive outward head impulses in both HIMP and SHIMP (8, 13, 14). Saccades became more grouped in patients with unilateral vestibulopathy after training with active head impulses in HIMP (15), but it was not determined whether predictability played a significant role. The effects of predictability on grouping of saccades in healthy subjects are not yet known.

The aim of this study was to identify differences in VOR gain and saccades in SHIMP between predictable and less predictable head movements in a group of healthy subjects. It was assumed that inward and active head movements could lead to higher prediction. Based on the previous studies, it was hypothesized that this higher degree of predictability could lead to a decrease in VOR gain values, a shorter saccadic latency, and higher grouping of saccades (7–9, 13–15). This hypothesis might imply that when SHIMP is applied in a predictable way (inward and/or active head impulses), specific measures should be taken to correct for the change in eye movement responses.

## Materials and Methods

### Study Population

This prospective study was performed in healthy subjects in Maastricht University Medical Center+ (MUMC+). The study lasted from October 2020 until April 2021. Subjects between 18 and 80 years old were included. Subjects were excluded if they met at least one of the following criteria: inability to see the point of fixation on the wall, inability to understand the examiner's instructions, severe physiological nystagmus, neck pathology or limited neck mobility, history of vestibular or neurological impairment or inner ear surgery, posture or gait abnormalities, severe hearing problems, prior use of alcohol at least 24 h before the study, or use of any tranquilizers, sedatives, or other vestibular suppressants at least 48 h before the study. A questionnaire was used to screen for the abovementioned criteria.

### Experimental Setup and Preparations

Examinations were performed using the ICS Impulse device (Natus, Taastrup, Denmark) with one camera focused on the pupil of the right eye. Each head impulse was applied by the same trained right-handed examiner (BV) and according to a previously published strict experimental setup (16, 17). The test subject was seated on a chair at a distance of 2 m from a wall. For HIMP, the target was fixated on this wall. During SHIMP, the target was projected on this wall by a head-mounted laser. In both cases, the target was at the level of the subject's eyes. The room was well lit to ensure a small pupil size required for an accurate pupil detection by the vHIT system. Shadows or light reflections onto the pupil were minimized (17). The head band of the goggles was tightly strapped. After the goggles were fixated, the rim of the goggles was adjusted so the eyelids were held back. The eye position was calibrated using the ICS Impulse two-point calibration (18). After successful calibration, the subject was instructed not to touch the strap, goggles, face, and head (17).

### Study Design

The protocol started in each participant with a HIMP session, to ensure an adequate functioning of the lateral semicircular canals. HIMP testing involved only passive outward horizontal impulses. Immediately after the HIMP, the SHIMP was performed using four conditions with different head movement patterns based on the previously described SHIMP protocol (5): passive head movements directed from the midline toward the side with gaze ended lateral (passive outward impulses); passive head movements directed from the side toward the midline with gaze ended central (passive inward impulses); active head movements directed from the midline toward the side with gaze ended lateral (active outward impulses); and active head movements directed from the side toward the midline with gaze ended central (active inward impulses). In order to control possible learning effects, the order of SHIMP conditions was randomized using the Latin square design (19). A summary of the test conditions is presented in Table 1.

**Table 1:** The conditions of the HIMP and SHIMP vHIT procedures.

	**Head direction**	
**Movement type**	**Inward**	**Outward**
Active	SHIMP	SHIMP
Passive	SHIMP	HIMP and SHIMP

*HIMP, head impulse paradigm; SHIMP, suppression head impulse paradigm; vHIT, video head impulse test*.

### Video Head Impulse Test Procedure

Subjects were instructed to keep their eyes wide open, to fixate on the target, and to not blink during testing. Before start of the official testing, slow horizontal sinusoidal head movements were given in order to assess neck stiffness and to give final instructions. In case of significant neck muscle tension during head impulses, the subject was excluded from the study. After the first six HIMP impulses, traces were analyzed in order to check for possible calibration problems or distinct artifacts. If no calibration problems or artifacts were observed, the official testing started.

During passive head impulses (HIMP and SHIMP), the examiner stood behind the subject with both hands on top of the head, holding it firmly without touching the strap or goggles. A head pitch between 0° and 15° downward was maintained. The head impulses comprised fast (peak velocity > 120°/s) horizontal rotational head movements with a small amplitude (±15°), unpredictable in timing and direction (only for outward). After each impulse, the participant's head was slowly moved back to the starting position by the examiner. Active head impulses (only SHIMP) were performed by the subjects themselves. They were asked to make rapid horizontal head rotations with the same velocity and amplitude as passive head impulses (8). A minimum of 10 impulses accepted by the device software were delivered to each side in each test condition. After every eight impulses, a small break was planned, so the subject could blink and relax for a short moment. The examiner repeated the instructions after each break to ensure optimal compliance.

### Data Cleaning

Head and eye velocity traces were exported and further processed using a custom-made software written in Python v.3.7.

The traces were automatically removed by custom-made software when (20, 21) head impulse bounce was more that 50% of the peak head velocity; head velocity never crossed zero after peak head velocity; head velocity was lower than 120°/s; mean head velocity calculated in the interval of 80 ms prior and 120 ms after peak head velocity was not in the range of the mean ± 3SD calculated for these means per subject, side, and test condition. After this procedure, the traces were manually inspected and removed based on consensus among three authors (RB, BV, and DS) if one of the following artifacts were present: the eye led the head; multiple head velocity peaks; an eye movement in the opposite direction of the expected VOR; oscillations not qualified as saccades; and the head velocity curve was not bell-shaped (4, 20, 21).

It should be noted that 120°/s was chosen as the minimum peak head velocity. This lower velocity allowed to collect enough data, since some subjects had difficulty to consistently reach high peak head velocities. This velocity was shown to be adequate for reliably testing VOR gain in children and adolescents, in which reaching high head velocities might also not always be feasible (22).

### Data Analysis

The onset of head movement was defined at the point of 60 ms before peak head acceleration. The offset of head movement was defined at the point where head velocity returned to zero. Timing of the peak head velocity was calculated related to the onset of the head movement.

VOR gain was used as the primary outcome measure. VOR gain for HIMP and SHIMP was calculated by the custom-made software (4) using the area under the curve method within the interval between head onset and offset (23). Both eye and head traces were desaccaded first before VOR gain was calculated. No interpolation was applied. Only data of subjects with mean gain values in HIMP ≥0.8 were used for the analysis (24).

Latency of the first saccade and the degree of grouping regarding timing (global PR-score, further in the text PR-score (25)) of all saccades were used as secondary outcome measures. The PR denomination does not have any mathematical or scientific significance (25). A custom-made algorithm was applied to extract saccades in SHIMP with as much accuracy as possible (4). Every saccade was verified by visual inspection by two of the authors (BV and DS). Saccades were included when (1) they occurred after head impulse onset, (2) they had a magnitude of more than 60°/s, and (3) their peak velocity was recorded. Erroneously detected saccades were manually excluded. Latency (in milliseconds) and the degree of grouping (PR-score) of the included saccades were extracted from the first 10 artifact-free traces. Saccade latency was related to the onset of the head impulse (4, 14). Only latency of the first saccade of each impulse was determined. The PR-score was calculated using the method originally implemented in the MATLAB open-source script named HITCal (25). For short, the PR-score is the weighted arithmetic mean of the variation coefficients of the first- and second-order saccades with the weights of 0.8 and 0.2, respectively. Two corrections are applied: the PR-score value is limited to 100 and in case when the PR-score is over 35, and the weight for the variation coefficient of the second-order saccades is reduced in the arithmetic mean inversely to their number (25).

### Statistical Analysis of Peak Head Velocities

Mean peak head velocity was calculated per subject and test condition. In order to account for a possible effect of head velocity on gain differences, two analyses were performed. First, means of the mean peak head velocities were compared in each pair of the test conditions using the paired *t*-test. Second, for each significant difference (separately for each side), a linear regression model was fitted with the difference of mean gain as the dependent variable and the difference of mean peak head velocity as the independent variable. These differences were calculated as follows: for active and passive head impulses, they were calculated as outward value minus inward value; for outward and inward head impulses, they were calculated as passive value minus active value. The α-level was set on 0.05. All p-values were Bonferroni corrected for multiple comparisons.

### Statistical Analysis of Main Outcomes

Mean age with a standard deviation was calculated for the tested group. Mean of the outcome measures (for HIMP and SHIMP: gain; only for SHIMP: latency of the first saccade and PR-score) was calculated per subject for each side and test condition. Mean with a 95% confidence interval was calculated for the means of the outcome measures.

Since all subjects produced at least one saccade in all SHIMP impulses, there were no missing data regarding latencies of the first saccade and PR-score. To analyze the effect of head movement type (active and passive), head direction (inward and outward), and side (left and right) on each outcome measure, three three-way repeated-measures ANOVAs (RANOVA) were fitted. Movement type (active and passive), head direction (inward and outward), and side (left and right) were set as the two-level within-subject factors including their two- and three-way interactions. The corresponding outcome measure (gain, latency, and PR-score) was set as the dependent variable. In case of statistical significance of the two-way interaction, two-way RANOVAs were fitted per each unique level of the corresponding factors. If in this model, a two-way interaction was significant, the paired *t*-test was used to evaluate pairwise comparisons between levels of the corresponding factors. The α-level was set on 0.05. The p-values were Bonferroni corrected for multiple comparisons.

#### Preliminary Statistical Results for Main Outcome Measures

For the three-way RANOVA with gain as the dependent variable, a two-way interaction between side and movement type was significant [F(1,56) = 4.38, I = 0.041], and a two-way interaction between movement type and head direction was significant [F(1,56) = 63.49, *p* < 0.001]. Therefore, 6 two-way RANOVAs were fitted.

For the three-way RANOVA with latency as the dependent variable, a two-way interaction between side and movement type was significant [F(1,56) = 5.43, *p* = 0.023], and a two-way interaction between side and head direction was significant [F(1,56) = 6.10, *p* = 0.017]. Therefore, 6 two-way RANOVAs were fitted.

For the three-way RANOVA with PR-score as the dependent variable, a two-way interaction between movement type and head direction was significant [F(1,56) = 7.08, *p* = 0.0]. Therefore, 4 two-way RANOVAs were fitted.

#### Additional Statistical Analysis of Gain Calculated by the Device Software

Gain values for each impulse were exported from the ICS Impulse software (vHIT software), which calculates gain using the same algorithm as the custom-made software (26). Mean gain values derived by both programs were compared per side and test condition using the paired *t-*test. The α-level was set on 0.05. The p-values were Bonferroni corrected for multiple comparisons. All statistical analyses were performed in R v.4.0.3 (R Foundation for Statistical Computing, Vienna, Austria) and SPSS Statistics v27 (SPSS Inc., Chicago, IL, USA).

### Ethical Considerations

This study was performed in accordance with the guidelines outlined by Dutch legislation. According to the Medical Research Involving Human Subjects Act (WMO), ethical approval was not required, since the purpose of this study was to validate our own system and to obtain the normative values. Written informed consent for participation and publication of these results was obtained from all subjects.

## Results

### Characteristics of Healthy Volunteers

**Figure 1:**
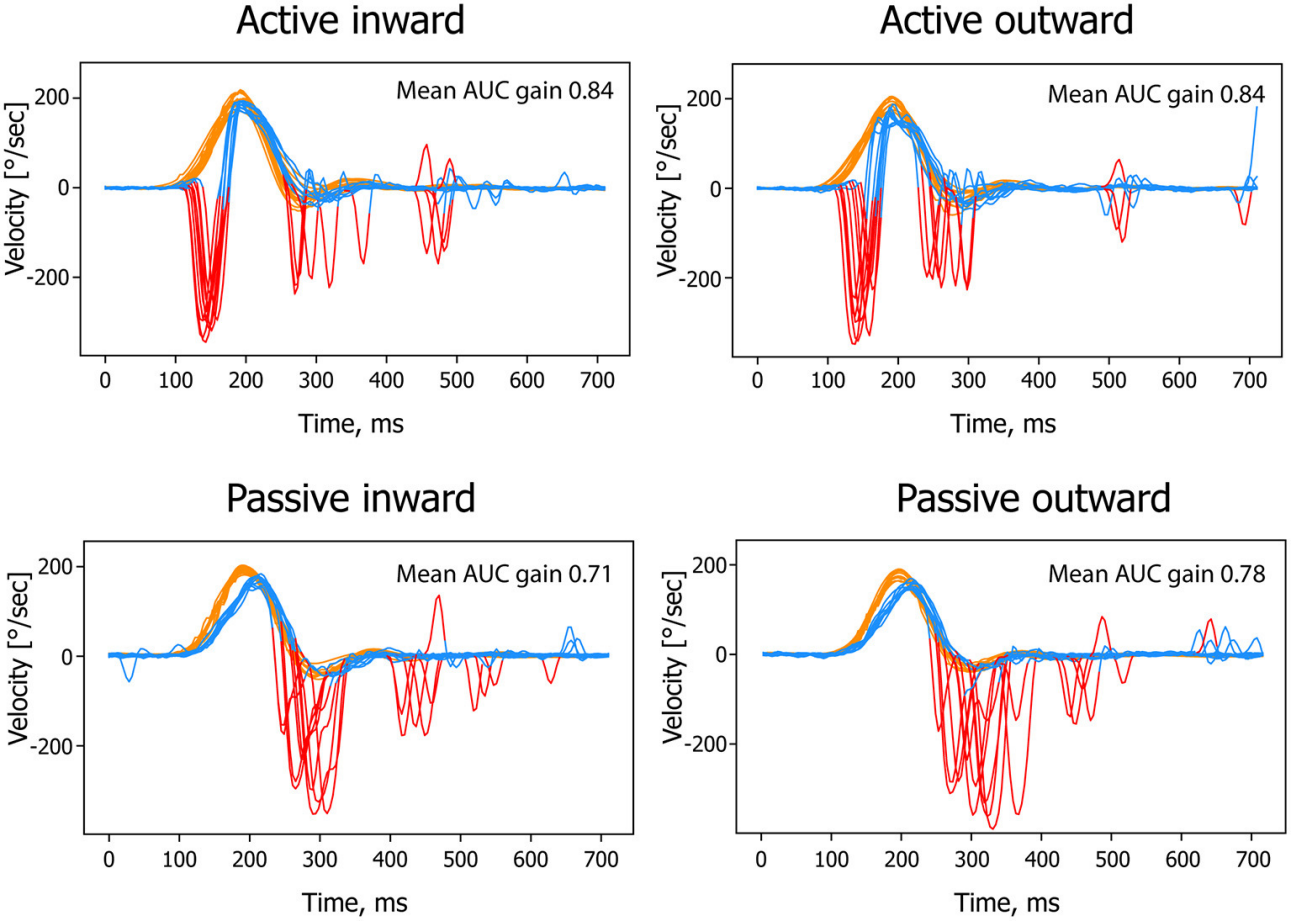
An example of SHIMP vHIT traces for all tested conditions (left side) in one test subject. SHIMP, suppression head impulse paradigm; vHIT, video head impulse test; AUC, area under curve.

Sixty-two healthy subjects were recruited, whose characteristics are presented in Table 2. Five subjects were excluded: two subjects were excluded due to pupil detection problems; one subject was excluded due to inadequate neck relaxation, which compromised the collection of appropriate head impulses; and two subjects were excluded due to <10 valid impulses per side. In total, vHIT data of 57 subjects were included, containing 10.440 impulses, of which 9.983 (96%) were free of artifacts. Since for each subject the first 10 artifact-free traces of each side per condition were included in the analysis, a total of 5.700 impulses were used for statistical analysis. All test subjects showed a mean VOR gain ≥0.8 when tested with passive outward HIMPs (Table 2). An example of horizontal SHIMP vHIT traces for all tested conditions in one subject is presented in Figure 1.

**Table 2:** Characteristics of the study population.

N	57
Male	26
Female	31
Mean age (years)	26 ± 3
Head impulse paradigm mean VOR gain (left)	0.92 ± 0.07
Head impulse paradigm mean VOR gain (right)	0.99 ± 0.08

*Age in years ± standard deviation. VOR gain ± standard deviation. Head impulse paradigm VOR gain is calculated for passive outward head impulses*.

*VOR, vestibular ocular reflex*.

### Vestibulo-Ocular Reflex Gain, Latency of the First Saccade, and Global PR-Score in Suppression Head Impulse Paradigm Conditions

**Figure 2:**
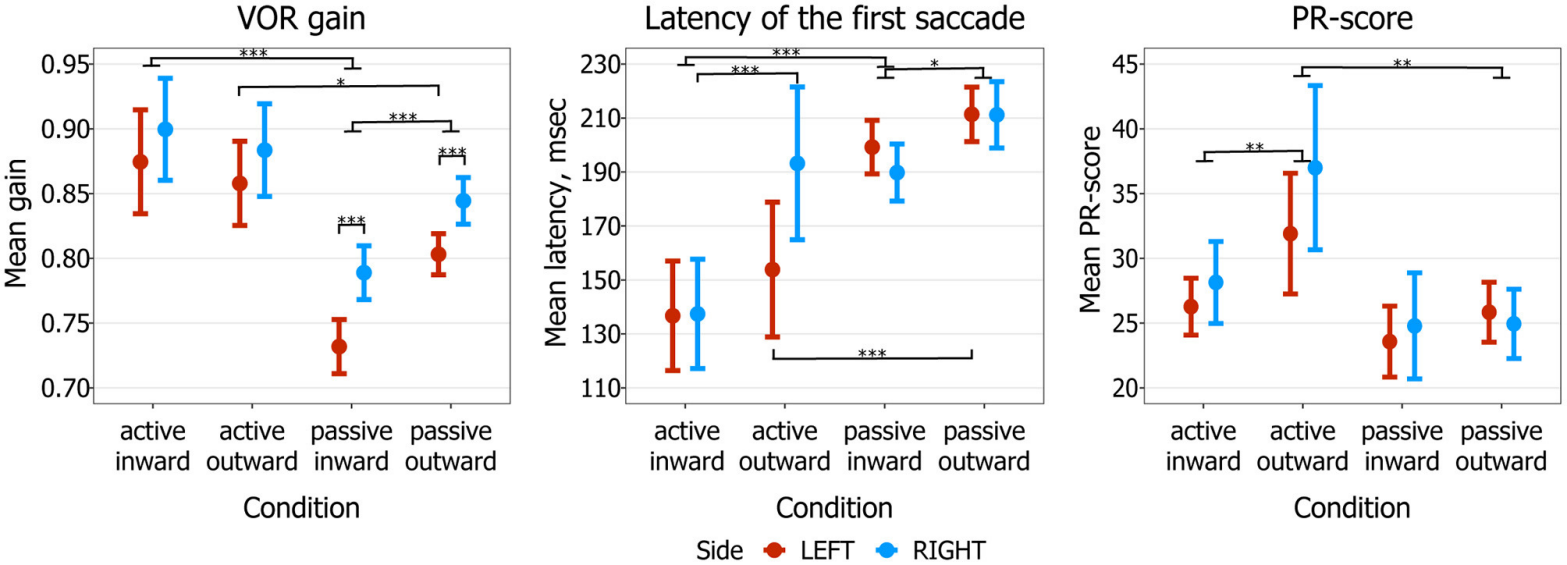
SHIMP mean values with corresponding 95% confidence intervals, calculated for mean VOR gain, mean latency of the first saccade, and mean PR-score. ^*^p < 0.5, ^**^p < 0.01, ^***^p < 0.001. SHIMP, suppression head impulse paradigm; VOR, vestibulo-ocular reflex.

For each tested SHIMP condition, means of VOR gain, latency, and PR-score are presented in Figure 2 and Table 3.

**Table 3:** Mean gain, mean latency of the first saccade, and mean PR-score with corresponding standard deviation (SD) and 95% confidence intervals (CI) for each combination of movement type, head direction, and side.

			**Gain**			**Latency, ms**			**PR-score**		
**Movement type**	**Head Direction**	**Side**	**Mean**	**SD**	**95% CI**	**Mean**	**SD**	**95% CI**	**Mean**	**SD**	**95% CI**
Active	Inward	Left	0.87	0.15	(0.83, 0.91)	137	75	(116, 157)	26	8	(24, 28)
Active	Inward	Right	0.90	0.15	(0.86, 0.94)	137	76	(117, 158)	28	12	(25, 31)
Active	Outward	Left	0.86	0.12	(0.83, 0.89)	154	94	(129, 179)	32	18	(27, 37)
Active	Outward	Right	0.88	0.14	(0.85, 0.92)	193	107	(165, 221)	37	24	(31, 43)
Passive	Inward	Left	0.73	0.08	(0.71, 0.75)	199	37	(189, 209)	24	10	(21, 26)
Passive	Inward	Right	0.79	0.08	(0.77, 0.81)	190	40	(179, 200)	25	15	(21, 29)
Passive	Outward	Left	0.80	0.06	(0.79, 0.82)	211	38	(201, 221)	26	9	(24, 28)
Passive	Outward	Right	0.84	0.07	(0.83, 0.86)	211	46	(199, 223)	25	10	(22, 28)

*Mean latency is in milliseconds*.

#### Vestibulo-Ocular Reflex Gain

Regarding movement type, mean gain was significantly higher in active than in passive head impulses in inward direction (*p* < 0.001). The same effect was observed in outward direction, but only for the left side (*p* = 0.024). Regarding head direction, mean gain was significantly higher in outward than in inward passive head impulses, regardless of side (*p* < 0.001). No significant difference was observed between inward and outward active head impulses. Regarding side, mean gain was significantly higher to the right than to the left in passive inward and outward head impulses (*p* < 0.001).

#### Latency of the First Saccade

Regarding movement type, mean latency of the first saccade was significantly shorter in inward active than in inward passive head impulses, regardless of side (*p* < 0.001). The same effect was observed in outward direction, but only for the left side (*p* < 0.001). Regarding head direction, mean latency was significantly shorter in passive inward than in passive outward head impulses, regardless of side (*p* = 0.012). The same effect was observed in active head impulses, but only for the right side (*p* < 0.001). Regarding side, mean latency was only significantly different between active outward left and right head impulses (lower in left, *p* = 0.006), but this effect became insignificant after the Bonferroni corrections (*p* = 0.144).

#### PR-Score

A significant difference in mean PR-score between active and passive head impulses was only observed in outward impulses, where it was higher in active head impulses regardless of side (*p* = 0.004). Furthermore, regarding head direction, mean PR-score was only significantly higher in active outward than in active inward impulses, regardless of side (*p* = 0.004). No significant differences were observed between sides in all tested conditions.

### Factors That Could Influence Main Outcome Measures

#### Differences in Head Velocities Between Tested Conditions

Mean peak head velocities and their latencies of each SHIMP condition are shown in Supplementary Table 1. The maximum difference in mean peak head velocities between conditions was 41°/s (active inward impulses to the left vs. passive outward impulses to the left). Mean peak head velocities were significantly lower for outward (both passive and active) and passive (both inward and outward) head impulses regardless of side (*p* ≤ 0.009). Eight linear models were fitted to assess the influence of the difference in mean peak head velocity on the difference in mean gain (outward minus inward and passive minus active, one per side). No significant effect was found in any pairs of the test conditions (*p* ≥ 0.32).

#### Difference in Vestibulo-Ocular Reflex Gain Calculation Between the Custom-Made Software and Video Head Impulse Test Device

Mean VOR gain values significantly differed in all SHIMP conditions between the custom-made software and the vHIT device (*p* < 0.001). The vHIT software calculated lower VOR gains in all test conditions (both sides), with the lowest values in active inward head impulses (Supplementary Figure 1).

## Discussion

This study compared VOR outcome measures of SHIMP between less predictable head movements (passive and outward) and more predictable head movements (active and inward). It was shown that in more predictable inward impulses, gain was lower than in outward impulses, but only for the passive head movements. The latency of the first compensatory saccades was shortened in all more predictable conditions. No significant influence of predictability was observed on grouping of the saccades.

The more predictable inward passive head movements demonstrated lower VOR gains than did the less predictable outward passive head impulses. This is congruent with the hypothesis and with previous literature (7, 9). Possible contributing factors are decreased alertness, less contraction of cervical muscles, and better VOR suppression due to the predictability during inward head impulses, leading to lower VOR gains (5–7, 9, 27–30). Predictability was also found to decrease the translational VOR gain (31). Although head velocities differed between test conditions in this study, it is less likely that these different head velocities contributed to the different VOR gains found in inward passive and outward passive impulses. After all, the variation in mean peak head velocities between passive inward and outward head impulses was little (10°/s for both sides), and statistical analysis demonstrated no effect of head velocity difference on VOR gain difference (32). Therefore, this study seems to support that higher predictability of head impulses leads to a lower VOR gain in SHIMP. Nevertheless, this VOR gain difference is relatively small (<0.1) and might not have any clinical consequences (7).

**Figure 3:**
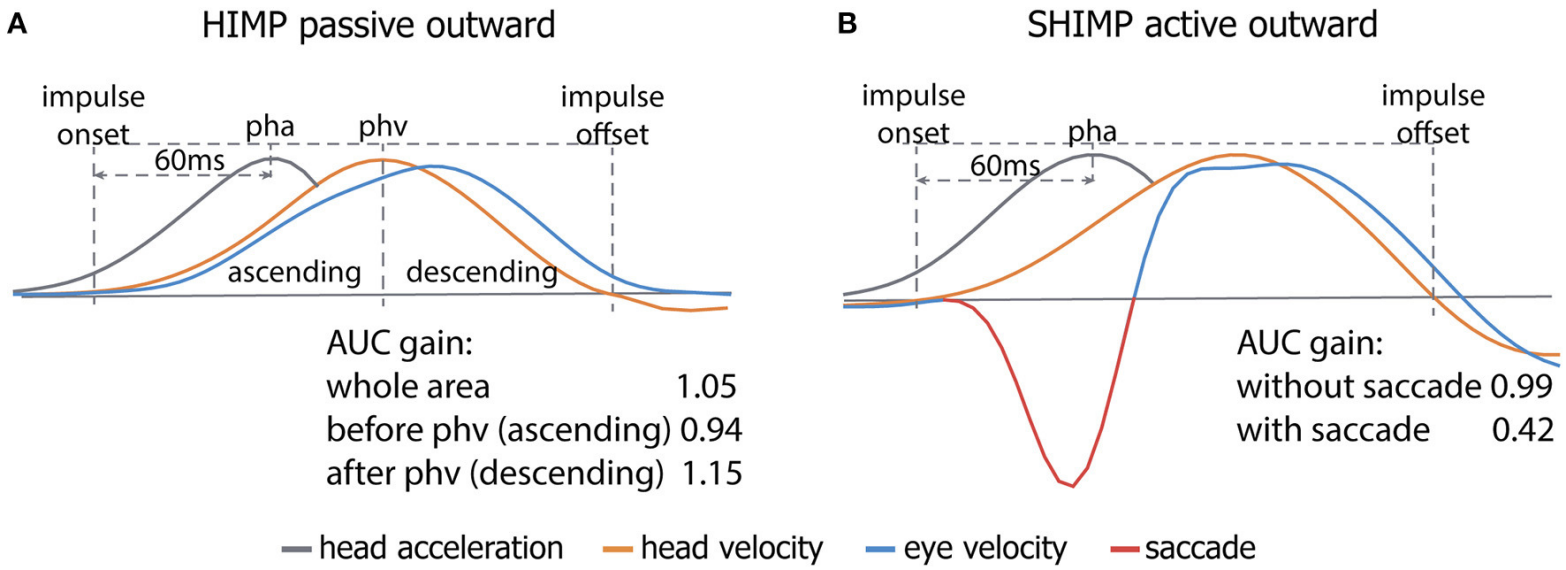
A schematic overview of the area under the curve (AUC) gain calculation method and its vulnerability to calculating gain from the ascending vs. descending phase **(A)** and the presence of an early saccade **(B)**. Due to the physiological delay of the vestibulo-ocular reflex (VOR), the AUC of the ascending phase of the eye response is lower than that of the descending phase, leading to an inherent lower gain calculated by the AUC method **(A)**. Not detecting the early saccade (red part of the eye velocity trace; **(B)**) by the video head impulse test (vHIT) system leads to a lower gain since the AUC of the saccade is subtracted from the remaining eye response (blue part of the trace; **(B)**). Legend: pha, peak head acceleration; phv, peak head velocity.

However, in contrast to the hypothesis suggested earlier, SHIMP VOR gain was significantly higher in the more predictable active head impulses than in the less predictable passive head impulses. This was previously also described in HIMP (8). Nevertheless, this does not directly imply that predictability leads to a higher gain in active SHIMP head impulses. After all, when comparing active head impulses with passive head impulses in SHIMP, gain calculation using the “area under the curve method” is compromised by early saccades that mainly occur during active head impulses. This results in a less reliable comparison between active and passive head impulses. Background of this phenomenon is that during active head impulses, 35% of the subjects in this study produced large (400°/s) saccades before the mean timing of peak head velocity, while during passive head impulses, saccades were predominantly produced after mean peak head velocity timing. Since saccades were eliminated from the traces without interpolation, gain for active head impulses was often based on the descending phase of the VOR curve (see Figures 1, 3), and gain for passive head impulses was often based on the ascending phase of the VOR curve (33). Gain calculated from the ascending phase of the VOR curve is expected to be lower than gain calculated from the descending phase of the VOR curve due to the fact that the VOR is physiologically delayed by on average 8–9 ns (see Figure 3A). Therefore, it cannot be reliably stated that active head movements demonstrate a higher gain related to predictability: the gain calculation method might also play a significant role (33, 34). Given the influence of early saccades on gain calculation, attention should be paid when comparing gain between active and passive head movements in future studies. These early saccades affect not only gain calculated by the area under the curve method but also other methods like instantaneous or regression gain. A possible solution to calculate and compare VOR gains could be to only include time points that are present in all impulses, after the desaccading process. This needs to be investigated in future trials.

Furthermore, higher mean peak head velocities were accompanied by higher mean gains in active head impulses. This is in contrast to previous studies, in which lower VOR gains were found with increasing head velocities in healthy subjects (HIMP, outward passive head impulses) (35–37). Again, this might be related to the influence of using ascending and descending phases during gain calculation.

SHIMP VOR gain values calculated by the vHIT software were significantly lower than those calculated by the custom-made software, although low VOR gains were not expected in this healthy population (HIMP gain > 0.8). This finding highlights the fact that VOR gain outcomes are very sensitive to pre-processing. Since both calculation methods are based on the same area under the curve method using the same interval, differences in gain values were most likely related to the desaccading process. Erroneously including an early saccade in the gain calculation process leads to a lower gain since its area under the curve is subtracted from that of the VOR (see Figure 3B). Although in some vHIT systems the minimal latency of saccades can be defined to detect saccades, one still need to be very cautious when letting the default software automatically process the traces. Manual inspection is still required.

Regarding the gain asymmetry observed in passive head impulses between sides, probably the main factor that could contribute to this finding is the side on which the camera was placed (right side) (35–40). In active head impulses, no significant difference was demonstrated between sides. A higher degree of predictability might mask this asymmetry.

The latency of the first saccade was shorter in the more predictable head movements, like in active inward head impulses when compared with passive inward head impulses, and in passive inward head impulses when compared with passive outward head impulses. Early saccades in voluntary head movements are well known in literature: for example, subjects produce earlier saccades if they are informed about the direction of the next impulse (14). Early saccades are thought to have a central and cervical-reflex origin (41–46). Their aim is to shift gaze toward the visual target and are usually followed and complemented by the VOR and smaller corrective saccades (8, 13). In case of a deficient VOR, they can facilitate improvement of dynamic visual acuity (47, 48). It should be noted that early saccades can precede head movements (42). In this study, this was also found in six subjects during inward and outward active head impulses. Such early saccades can be “invisible” for the vHIT software, since saccade detection often only starts at least after the head impulse onset (18, 49). This phenomenon should therefore be taken into account during the gain calculation process of active head impulses.

The present study did not find any significant differences in the level of saccade grouping among different head movement paradigms, except for a significantly higher PR-score in active outward head impulses. This could mean that although subjects produced earlier saccades in case of active and inward impulses, the saccades were grouped approximately at the same level. However, this does rule out the effect of the predictability on saccade grouping. Further studies of the influence of predictability on saccade grouping in healthy subjects are required.

### Limitations of the Study

The main limitation of this study is a significantly hindered comparison of the gain values between active and passive head movements. In addition, the effect of age on consequences of predictability of head movements in SHIMP could not be determined due to relatively small age structure. However, this is less likely of relevance since the gain is known to be stable until at least 70 years (36). Furthermore, as a result of study design (using SHIMP in order to be able to study saccadic responses in healthy subjects), not all head movement paradigms were tested in HIMP. It therefore cannot be determined whether these findings in SHIMP can be generalized to HIMP.

## Conclusion

For SHIMP, a higher predictability in head movements lowered gain only in passive impulses and shortened latencies of compensatory saccades overall. For active impulses, gain calculation was affected by short-latency compensatory saccades, hindering reliable comparison with gains of passive impulses. Predictability did not substantially influence grouping of compensatory saccades.

## Data Availability Statement

The raw data supporting the conclusions of this article will be made available by the authors, without undue reservation.

## Ethics Statement

Ethical review and approval was not required for the study on human participants in accordance with the local legislation and institutional requirements. The patients/participants provided their written informed consent to participate in this study.

## Author Contributions

BV, LV, and TV carried out the experiment. MJ, BV, and DS performed the statistical analysis. DS and BV made the software for analysis. DS, BV, and RV wrote the manuscript. MP, LV, NG, AP, VV, and HK critically revised the manuscript. All authors participated in the design of the experimental protocol and analysis.

## Conflict of Interest

The authors declare that the research was conducted in the absence of any commercial or financial relationships that could be construed as a potential conflict of interest.

## Publisher's Note

All claims expressed in this article are solely those of the authors and do not necessarily represent those of their affiliated organizations, or those of the publisher, the editors and the reviewers. Any product that may be evaluated in this article, or claim that may be made by its manufacturer, is not guaranteed or endorsed by the publisher.
