# Supplementary material for: The Effect of Different Head Movement Paradigms on Vestibulo-Ocular Reflex Gain and Saccadic Eye Responses in the Suppression Head Impulse Test in Healthy Adult Volunteers
Source: Front Neurol. 2021 Sep 22;12:729081. doi: 10.3389/fneur.2021.729081 (PMC8492894; doi:10.3389/fneur.2021.729081)
Supplement: Supplementary file 1 [file Data_Sheet_1.docx]

**Supplementary material**

Table 1. Mean peak head velocities and their timings calculated for each SHIMP condition

| Movement  type | Head  direction | Side | **Peak head velocity, deg/s** | | | | **Timing, msec** | | | |
| --- | --- | --- | --- | --- | --- | --- | --- | --- | --- | --- |
|  |  |  | Mean | SD | Median | 1^st^\|2^nd^ quartiles | Mean | SD | Median | 1\|2^nd^ quartiles |
| Active | Inward | LEFT | 208 | 13 | 205 | 176\|237 | 104 | 13 | 102 | 96\|110 |
| Active | Inward | RIGHT | 196 | 13 | 192 | 167\|222 | 106 | 13 | 106 | 98\|114 |
| Active | Outward | LEFT | 188 | 14 | 183 | 157\|214 | 105 | 14 | 102 | 96\|111 |
| Active | Outward | RIGHT | 190 | 14 | 188 | 160\|216 | 105 | 14 | 102 | 98\|110 |
| Passive | Inward | LEFT | 176 | 13 | 177 | 161\|190 | 107 | 13 | 107 | 98\|115 |
| Passive | Inward | RIGHT | 186 | 11 | 187 | 170\|202 | 102 | 11 | 102 | 94\|110 |
| Passive | Outward | LEFT | 167 | 13 | 166 | 153\|180 | 105 | 13 | 102 | 97\|114 |
| Passive | Outward | RIGHT | 176 | 11 | 174 | 160\|192 | 103 | 11 | 102 | 98\|110 |


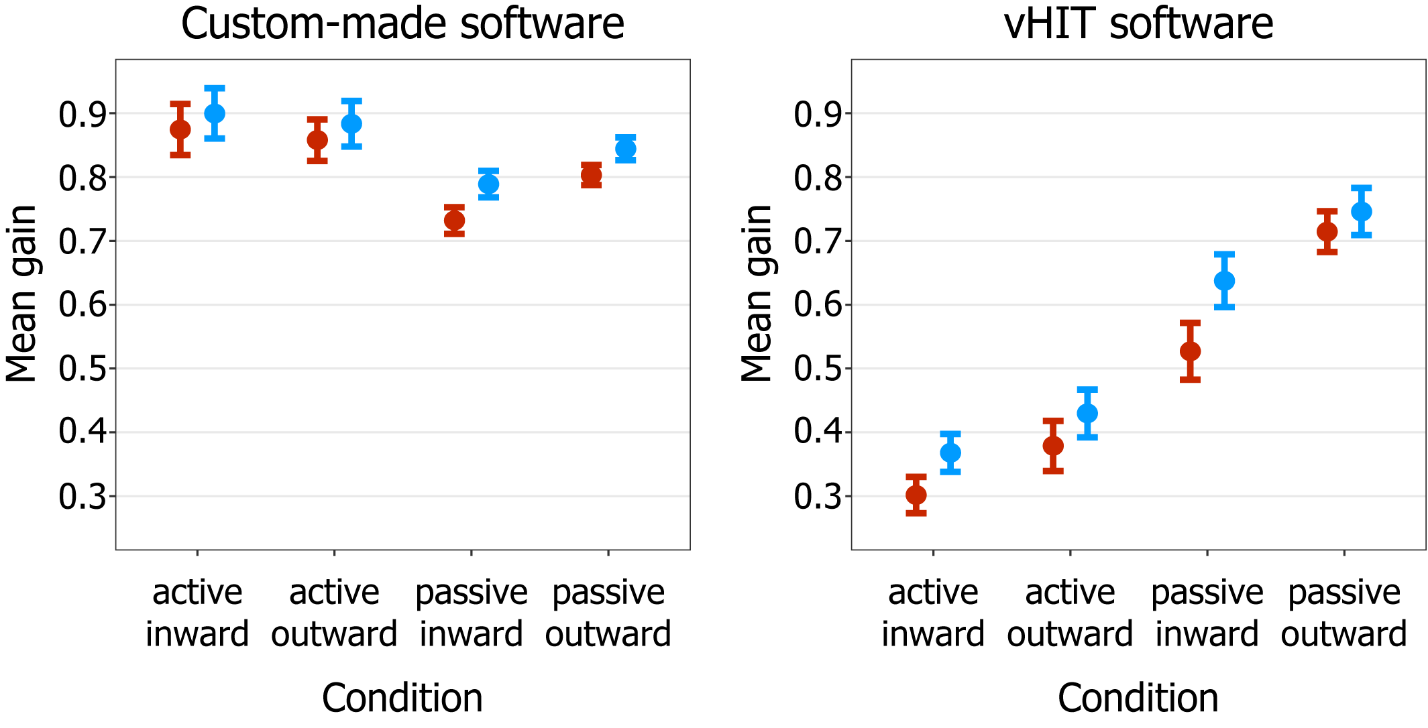


*Figure 1. SHIMP mean VOR gains with corresponding 95% confidence intervals, as calculated by the custom-made software (modified from Figure 2) and the vHIT device software.*
